# Intestinal Barrier Breakdown and Mucosal Microbiota Disturbance in Neuromyelitis Optical Spectrum Disorders

**DOI:** 10.3389/fimmu.2020.02101

**Published:** 2020-09-02

**Authors:** Chunping Cui, Sha Tan, Li Tao, Junli Gong, Yanyu Chang, Yuge Wang, Ping Fan, Dan He, Yiwen Ruan, Wei Qiu

**Affiliations:** ^1^Multiple Sclerosis Center, Department of Neurology, The Third Affiliated Hospital of Sun Yat-sen University, Guangzhou, China; ^2^Department of Gastroenterology, The Third Affiliated Hospital of Sun Yat-sen University, Guangzhou, China; ^3^Department of Colorectal Surgery, The Sixth Affiliated Hospital of Sun Yat-sen University, Guangzhou, China; ^4^Guangdong Provincial Key Laboratory of Colorectal and Pelvic Floor Diseases, The Sixth Affiliated Hospital of Sun Yat-sen University, Guangzhou, China; ^5^Department of Pathology, The Third Affiliated Hospital of Sun Yat-sen University, Guangzhou, China; ^6^GHM Institute of CNS Regeneration, Jinan University, Guangzhou, China; ^7^Co-innovation Center of Neuroregeneration, Nantong University, Nantong, China

**Keywords:** neuromyelitis optica spectrum disorders, tight junction, intestinal barrier, mucosa microbiota, intestinal inflammation

## Abstract

**Background and Purpose:**

The mechanism underlying the pathology of neuromyelitis optica spectrum disorders (NMOSD) remains unclear even though antibodies to the water channel protein aquaporin-4 (AQP4) on astrocytes play important roles. Our previous study showed that dysbiosis occurred in the fecal microbiota of NMOSD patients. In this study, we further investigated whether the intestinal barrier and mucosal flora balance are also interrupted in NMOSD patients.

**Methods:**

Sigmoid mucosal biopsies were collected by endoscopy from six patients with NMOSD and compared with samples from five healthy control (HC) individuals. These samples were processed for electron microscopy and immunohistochemistry to investigate changes in ultrastructure and in the number and size of intestinal inflammatory cells. Changes in mucosal flora were also analyzed by high-throughput 16S ribosomal RNA gene amplicon sequencing.

**Results:**

The results from bacterial rRNA gene sequencing showed that bacterial diversity was decreased, but *Streptococcus* and *Granulicatella* were abundant in the colonic mucosa specimens of NMOSD patients compared to the HC individuals. The intercellular space between epithelia of the colonic mucosa was wider in NMOSD patients compared to the HC subjects (*p* < 0.01), and the expression of tight junction proteins [occludin, claudin-1 and zonula occludens-1 (ZO-1)] in NMOSD patients significantly decreased compared to that in the HC subjects. We also found numerous activated macrophages with many inclusions within the cytoplasm, mast cells with many particles in their cytoplasm, and enlarged plasma cells with rich developed rough endoplasmic reticulum in the lamina propria of the mucosa of the patients with NMOSD. Quantitative analysis showed that the percentages of small CD38+ and CD138+ cells (plasma cells) were lower, but the percentage of larger plasma cells was higher in NMOSD patients.

**Conclusion:**

The present study demonstrated that the intestinal barrier was disrupted in the patients with NMOSD, accompanied by dysbiosis and inflammatory activation of the gut. The mucosal microbiota imbalance and inflammatory responses might allow pathogens to cross the damaged intestinal barrier and participate in pathological process in NMOSD. However, further study on the pathological mechanism of NMOSD underlying gut dysbiosis is warranted in the future.

## Introduction

Neuromyelitis optica spectrum disorders (NMOSD) is recognized as a distinct clinical entity from multiple sclerosis (MS) based on the disease-specific serum autoantibody aquaporin-4 (AQP4)-IgG. A recent study showed extensive homology between gut bacteria and AQP4 protein, implying molecular mimicry in the pathogenesis of the NMOSD (1).

The brain-gut axis involves multiple complicated connections among the gut microbiota, intestinal barrier and immune system in autoimmune diseases (2). A structurally and functionally intestinal barrier is fundamental to gut health. Any insults from the external environment including gut microbiota, toxins, drugs, and undigested food may undermine intestinal integrity and damage the intestinal barrier by triggering intestinal inflammation (3, 4). Since tight junction (TJ) proteins are the major components of the intestinal barrier, pathogens including bacteria, toxins, microbial products or undigested food may enter the lamina propria, leading to pathological consequences after the TJ proteins and epithelial barrier are breached (3).

Several studies (5, 6) have reported that fecal microbes may not accurately represent the engraftment colony in the gastrointestinal tract. The microbiota of the intestinal mucosa and feces and their relevance to diseases are different. For example, data from patients with inflammatory bowel diseases (5) or chronic constipation (6) suggested that evaluating the mucosal microbiota is better than evaluating the fecal microbiota. Recently, our group (7, 8) and others (9, 10) reported the occurrence of fecal microbiota disturbance in NMOSD. However, the roles of the mucosal microbiota and intestinal barrier in NMOSD remain unclear.

In the present study, we further investigated pathological changes in the colonic mucosa, including the intestinal barrier, mucosal flora and inflammatory response, in NMOSD patients.

## Materials and Methods

### Research Participants

Six Chinese NMOSD patients who fulfilled the criteria of Wingerchuk (11) and were seropositive for AQP4-IgG were consecutively enrolled from the Multiple Sclerosis Center. Five healthy controls (HC) were recruited from the Health Examination Center of The Third Affiliated Hospital, Sun Yat-sen University, from July 2019 to October 2019. The participants in the patient group had no history of intravenous methylprednisolone therapy or disease-modifying therapies for 6 months. The control group was matched for body mass index (BMI), age, and sex. Subjects were excluded if they consumed alcohol or tobacco or had consumed antibiotics or probiotics within the previous month. Furthermore, pregnant or lactating females and individuals who suffered from hepatitis, systemic autoimmune disease, carcinoma and gastrointestinal disease were also excluded. To reduce the effect of diet on the composition of the mucosal microbiota, subjects were included if they had an appropriate fat intake (fat calorie intake was no more than 35% of total calories) and did not consume peppery food or yogurt for the last 7 days. The severity of the NMOSD was assessed using the Expanded Disability Status Scale (EDSS) score, which was divided into three classes (<3, 3–5, >5). The patient cohort in this study was independent from our previously published cohorts (7, 8).

The present research was approved by the ethics committee of The Third Affiliated Hospital of Sun Yat-sen University. Written informed consent was obtained from the participants.

### Mucosal Specimen Collection

Previous studies (12) have reported no significant difference in the microbiota associated with ileal, cecal and rectal mucosa. Sigmoid mucosal biopsies were collected via endoscopy in the present study. A limited, prepped sigmoidoscopy was performed using a standard adult fibro-colonoscope to 20–25 cm from the anal verge. Biopsies were taken from pink mucosa without visible feces at the sigmoid colon approximately 20 cm from the anal verge and were either snap frozen at −80°C or processed with 2.5% glutaric dialdehyde, protein preservation solution (Kingmed Diagnostics, Guangzhou, China) and 10% formalin fixation solution in the endoscopy room.

### Analysis of Intestinal Microbiota

Bacterial DNA was extracted from colonic mucosal samples with the QIAamp Power Fecal DNA Kit (Qiagen, Germany) according to the manufacturer’s instructions. The bacterial DNA was amplified using barcoded primers that amplified the V3–V4 hypervariable region of the 16S rRNA gene (∼500 bp long). PCR products were examined on 2% (w/v) agarose gel and further purified using an E.Z.N.A. Gel Extraction Kit (Omega Biotek). Construction of sequencing libraries and paired sequencing (2 × 250 bp) were performed on an Illumina MiSeq platform at Biomarker Technologies Co., Ltd. (Beijing, China) according to standard protocols. Custom Perl and Bash scripts were used to demultiplex the reads and assign barcoded reads to individual samples. Reads were retained only when the sequence included a perfect match to the barcode and the V4 16S rRNA gene primers and were within the length expected for the V3–V4 variable region. The raw data were merged using FLASH (13). Sequences were quality filtered using Trimmomatic (14), and chimera sequences were removed using the UCHIME algorithm (15).

### Transmission Electron Microscopy and Data Analysis

After washing in phosphate buffered saline, samples were placed in osmium tetroxide for 2 h. Then, the samples were dehydrated in a series of ethanol solutions of increasing concentrations until 100%, infiltrated with propylene oxide, embedded in pure resin (Epon812, TED PELLA, United States) and solidified. Localization of the mucosa was achieved under an Olympus optical microscope (model: BX41, Olympus, Hamburg, Germany), and then one-micron-thick semi-thin sections were cut using a Leica ultrathin microtome (model: UC-7, Leica Microsystems, Wetzlar, Germany). After the tissue of a whole section was observed, ultrathin sections (50–70 nm) were cut on the same microtome. The ultrathin sections were stained with 2% uranium dioxide acetate (SPI Supplies, West Chester, PA, United States) and lead citrate (TED PELLA, United States). The experiments were performed in duplicate.

Images with higher minification (25,000×) were obtained under a transmission electron microscope (TEM) (JEM-1400 PLUS, Japan Electron Optics Laboratory Co., Ltd, Japan). Three to five images were selected from each individual. The intercellular spaces, including TJs, adherens junctions (AJs) and desmosomes (des) between two epithelial cells were measured by ImageJ (National Institutes of Health, United States, Version 1.51k). An intercellular space was defined as the gap from the border of one epithelium to the border of the adjacent epithelium, excluding the dense adherent structures. The measurements were performed by two researchers independently.

### Immunofluorescence Staining

Sigmoid colon mucosal specimens were postfixed in protein preservation fixative (Kingmed Diagnostics, Guangzhou, China) and then washed twice with protein preservation cleaning solution for 10 min each. Sections of 4 μm were cut from frozen mucosa on a cryostat (serial no. 0325; Thermo Fisher Scientific, Cheshire, United Kingdom) and fixed in 4°C acetone for 10 min. A circle was drawn around the sections on a slide by a Dako pen (code no. S2002; Dako, Glostrup, Denmark) to prevent the antibody from flowing out. Then, the sections were incubated for 40 min at 37°C with primary antibodies against zonula occludens-1 (ZO-1) (rabbit anti-ZO1 antibody 1:50, Abcam, ab96587), occludin (OCC) (rabbit anti-occludin antibody 1:50, Abcam, ab235986), and claudin-1 (CLA) (rabbit anti-claudin-1 antibody 1:50, Abcam, ab15098) separately. After washing in 0.01 M PBS 3 times for 5 min each, the sections were incubated with Alexa Fluor-conjugated secondary antibody (Goat Anti-Rabbit IgG H&L 1:400, Abcam, ab150077). Finally, the sections were covered with glycerine and a glass coverslip. All the steps were repeated twice.

Fluorescence images were taken using a Zeiss confocal microscope (LSM700, Zeiss, Germany) under the same conditions as the light microscope, and images in different channels were overlaid using Adobe Photoshop (v. CS3, Adobe, San Jose, CA, United States). Three to five images were selected from each specimen for quantitative analysis of immunofluorescent staining. The fluorescent densities (AODs) of OCC, CLA, and ZO-1 were measured using ImageJ under the same conditions by two researchers independently.

### Immunohistochemistry

All biopsy samples from the NMOSD patients and HC subjects were processed for immunohistochemical staining to detect antigens of CD3, CD20, CD38, CD68, and CD138. Blocks of colonic mucosa were fixed by formalin, embedded in paraffin and then cut into 4-μm-thick sections. After xylene dewaxing, gradient ethanol rehydration and high-pressure antigen retrieval, tissue sections were stained with the selected streptavidin–biotin–peroxidase (SP) staining method. Guided by the Novolink Detection Kit instructions (Leica Novocastra, Re7280-k, Germany), the following operations were carried out: all sections were treated with primary antibodies at 4°C overnight. These antibodies included anti-CD3 and anti-CD20 antibodies (1:200, from rabbit, Dako Denmark, Glostrup, Denmark) and anti-CD38 (1:400), anti-CD68 (1:800), and anti-CD138 (1:600) antibodies (from rabbit, Leica Microsystems, Deerfield, IL, United States). Then, these sections were incubated with biotin-conjugated anti-rabbit secondary antibody for 30 min at 37°C followed by the enzyme substrate 3′,3-diaminobenzidine tetrahydrochloride (DAB reagent kit, Re7163, Germany) for color development. Brown staining of CD3, CD20, CD38, CD68, and CD138 on the cell membrane was classified as positive staining. Negative controls were processed in the same manner without primary antibodies. These experiments were repeated twice.

Images were obtained under an Olympus microscope (model: BX43, Olympus, Hamburg, Germany) under the same conditions as the light microscope. Three to five pictures were selected from each specimen for quantitative measurement.

(1) The density of inflammatory cells: We employed five antibodies to detect activation of inflammation: CD3, a marker for T lymphocytes; CD20, a marker for B lymphocytes; CD38, which is expressed on B lymphocytes and plasma cells; CD68, a marker for monocytes and macrophages; and CD138, a marker for plasma cells. We counted the average number of inflammatory cells in the lamina propria in five representative high-power fields (HPFs, 400×), and the results were presented as the average number of cells/HPF.
(2) The size of inflammatory cells: We randomly selected 30–50 CD3-, CD20-, CD38-, CD68-, and CD138-positive cells from each specimen and measured the area of the cells at HPFs (400×) to assess cell functional status by ImageJ. Based on the data of the area, we classified these cells into five levels and performed comparisons among the different levels of the cells.

### Statistical Analysis

Statistical analysis was performed in SPSS (version 20.0, Armonk, NY, United States: IBM Corp) and GraphPad Prism 6.0 software (GraphPad Software Inc., San Diego, CA, United States). After passing equal variance testing, BMI, intercellular spaces, and the AODs of CLA, OCC, and ZO-1 were analyzed by the Mann–Whitney test. The parameters of the average number of cells/HPF in immunohistochemical staining, the age and BMI of the subjects, and sizes of cells were analyzed by *t*-tests. The data are presented as the mean ± SEM. α-diversity (Shannon-Wiener diversity index) was calculated based on the rarefied operational taxonomic units (OTUs). Principal coordinate analysis (PCoA) was coordinated from the weighted UniFrac distance, and the linear discriminant analysis (LDA) effect size (LEfSe) pipeline (16) and Metastats (17) were employed to differentially identify microbes that distinguished patients from HC subjects. The effective sequences were binned into OTUs using USEARCH software with a cut-off of 97% identity in 16S (18). A value of *p* < 0.05 was considered statistically significant in the compared groups.

## Results

### Information Collected From HC and NMOSD Subjects

Information regarding the number, sex, age, BMI, AQP4-IgG status, and disease severity of all subjects is presented in Table 1.

**TABLE 1:** Demographic and clinical features of the NMOSD and HC groups.

	NMOSD	HC	*p*-Value
***N***	**6**	**5**	
Female, *n* (%)	6 (100%)	5 (100%)	
Age, years	41.67 ± 14.95	36.40 ± 11.26	0.534
BMI, kg/m^2^	21.19 ± 3.49	20.95 ± 2.08	0.855
AQP4-IgG, *n* (%)	6 (100%)	–	
**EDSS score**			
<3	2 (33.33%)		
3–5	1 (16.67%)		
>5	3 (50.00%)		

*NMOSD, neuromyelitis optica spectrum disorders; HC, healthy control; BMI, body mass index; AQP4, aquaporin-4; EDSS, Expanded Disability Status Scale; IgG, immunoglobulin G.*

### Intestinal Mucosal Dysbiosis in NMOSD Patients

**FIGURE 1:**
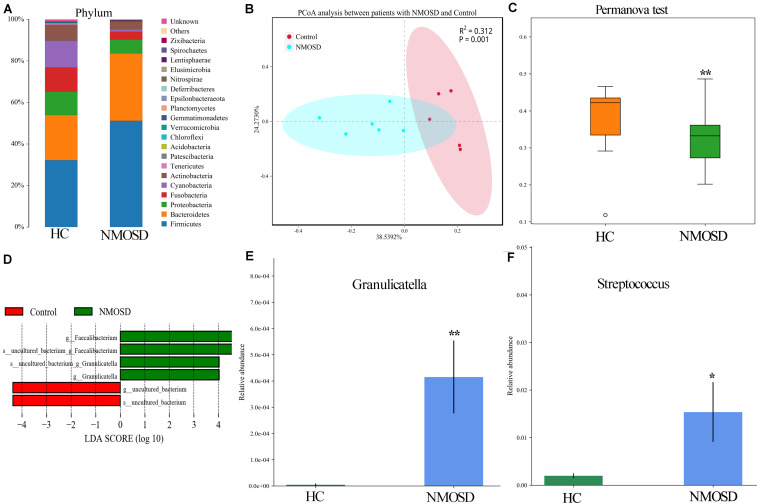
Histogram and scatter diagram showing the distribution characteristics of the intestinal microbiota among the two groups. **(A)** The distribution characteristics of the intestinal microbiota of the colonic mucosa originated from the same species at the phylum level. **(B)** The bacterial cluster from HC individuals was significantly distinguished from that of NMOSD patients by PCoA analysis. Each point represents the composition of the intestinal microbiota of one participant. **(C)** PERMANOVA showed that bacterial diversity was decreased in the NMOSD patients compared to the controls (*p* = 0.001 < 0.01). **(D)** LEfSe analysis showed that *Granulicatella* and *Faecalibacterium* were dominant in NMOSD specimens (green). **(E)** The relative abundance of *Granulicatella* in participants from the two groups (NMOSD vs HC; ^∗∗^*p* < 0.01, Metastats analysis). **(F)** Comparison of the relative abundance of *Streptococcus* among the two groups (NMOSD vs HC; ^∗^*p* < 0.05, Metastats analysis).

In the present study, a small piece of colonic mucosa specimen was obtained from each NMOSD patients to detect changes in intestinal microbiota using 16S rRNA sequencing. The results are shown in Figure 1. After applying strict trimming criteria to exclude low-quality reads, a total of 684,738 reads displaying acceptable quality were obtained, with an average of 62,249 reads per sample. Taxonomic classification at the phylum level revealed that the intestinal mucosal bacteria of these specimens mainly consisted of *Firmicutes*, *Bacteroidetes*, and *Proteobacteria* (Figure 1A). No significant difference in the biodiversity of the mucosal microbiota was detected by the Shannon index between in the healthy individuals and NMOSD patients (Supplementary Figure S1). However, based on the weighted UniFrac distance and PCoA, the bacterial cluster from HC subjects was significantly distinguished from that of NMOSD patients (Figure 1B). PERMONOVA analysis showed that bacterial diversity was decreased in the NMOSD specimens compared to controls (Figure 1C, *p* = 0.001 < 0.01, PERMANOVA). According to the LEfSe analysis, two bacteria at the genus level, *Granulicatella* and *Faecalibacterium*, were dominant in NMOSD patients (Figure 1D). Based on the data from the Metastats analysis on 40 bacteria at the genus level (Table 2), among the 20 bacteria that were increased in NMOSD patients (Table 2, orders 1–20), including some short chain fatty acids-producing bacteria (19–22) (*Faecalibacterium*, *Roseburia*, *Coprococcus*, *Ruminococcaceae*, *Lachnospira*), which have protective role in colonic inflammation, and some proinflammatory bacteria, such as *Granulicatella* (23), *Streptococcus* (24), *Proteus* (25), and *Desulfovibrio* (26). Among the 20 bacteria that were decreased in NMOSD patients, most are conventional intestinal bacteria, and many are beneficial bacteria such as *Rahnella* (27), *Lactococcus* (28), *Leptotrichia* (29) and *Turicibacter* (30) (Table 2, orders 21–40). *Granulicatella* and *Streptococcus*, two well-studied bacteria, were significantly increased in NMOSD patients compared with HC subjects (Figure 1E, *p* < 0.01 and Figure 1F, *p* < 0.05, respectively).

**TABLE 2:** Composition of microbiota genera in the NMOSD and HC groups by Metastats.

	Genus	Mean (HC)	Mean (NMOSD)	*p*-Value
1	Subdoligranulum*	0.003184604	0.020584006	0.002156863
2	Granulicatella	4.39E-06	0.000415129	0.002896359
3	Uncultured_bacterium_f_Lachnospiraceae	0.006679129	0.017050095	0.003753501
4	Coprococcus_1	0.000537436	0.003066943	0.004285714
5	Butyricicoccus	0.000268146	0.002462711	0.004445378
6	Phascolarctobacterium	0.000595199	0.014323617	0.004515406
7	Ruminococcaceae_UCG-003	0.000120348	0.004230241	0.005022409
8	Ruminococcaceae_UCG-002	0.00144715	0.013862003	0.008596639
9	Lachnospira	0.002455323	0.006297456	0.011980392
10	Sutterella	0.002118788	0.018046937	0.012263305
11	Faecalibacterium	0.024730502	0.113679063	0.020190476
12	Streptococcus	0.00195043	0.015309921	0.0212493
13	Proteus	4.11E-05	0.000140499	0.024745098
14	Roseburia	0.007092409	0.022230644	0.028347339
15	Lachnoclostridium	0.003638381	0.008673288	0.03380112
16	Christensenellaceae_R-7_group	0.000370886	0.001205755	0.034526611
17	Lachnospiraceae_UCG-008	0.000969102	0.006802506	0.034935574
18	Desulfovibrio	0.000416844	0.006562565	0.035347339
19	(Eubacterium)_ventriosum_group	6.29E-05	0.000491487	0.040605042
20	Ruminococcaceae_NK4A214_group	0.000519509	0.001508411	0.046557423
21	Staphylococcus	0.00290804	0.001022846	0.00259944
22	Sphingomonas	0.003204459	0.000767466	0.004492997
23	Leuconostoc	0.000862937	5.45E-06	0.01494958
24	Blastococcus	0.000160036	1.16E-05	0.015338936
25	Rahnella	0.000294684	6.37E-05	0.015672269
26	Lactococcus	0.002000652	0.000134881	0.015795518
27	Ruminiclostridium_9	0.001697474	0.000435464	0.015879552
28	Enterorhabdus	0.001494197	0.000124622	0.020246499
29	Leptotrichia	0.000500136	6.95E-06	0.020308123
30	Morganella	0.000476049	3.00E-05	0.022823529
31	Turicibacter	0.003200864	0.00093676	0.02427451
32	Faecalibaculum	0.015936691	0.000464709	0.026495798
33	Uncultured_bacterium_f_Gemmatimonadaceae	0.000439462	3.74E-05	0.029347339
34	Ruminiclostridium_6	0.001248236	1.73E-05	0.029383754
35	Dubosiella	0.033559273	0.001424643	0.036717087
36	Pseudaminobacter	0.000170509	2.82E-05	0.040487395
37	Candidatus_Solibacter	0.000986056	5.76E-05	0.042487395
38	Janthinobacterium	0.000439663	2.09E-05	0.045492997
39	Lachnospiraceae_UCG-006	0.004080315	0.000207736	0.046182073
40	Uncultured_bacterium_o_PLTA13	0.000424525	1.53E-05	0.049397759

*Upper section (1–20): bacteria were increased at the genus level in the NMOSD group. Lower section (21–40): bacteria were decreased at the genus level in the NMOSD group.

### The Width of Intercellular Spaces Was Increased in the Colonic Mucosa of NMOSD Patients

**FIGURE 2:**
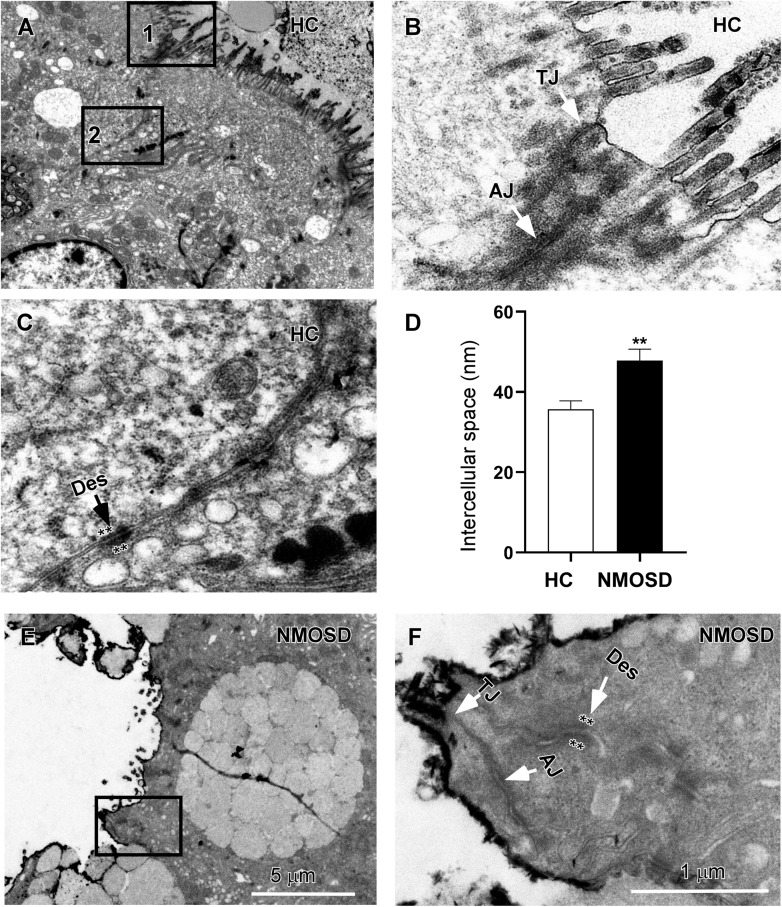
TEM photos and a histogram showing changes in intercellular spaces of the epithelium. **(A)** Low-power photos showing the intercellular space of the epithelium in HC group. **(B)** High-power photos showing the tight junction (TJ) from square box 1 of panel **(A)** and the adherens junction (AJ) from square box 2 of panel **(B)**. **(C)** High-power photos showing the desmosome (des) with dense and coarse cytoplasmic fibrils attaching on either side of the junction (asterisks). **(E)** Low-power photos showing the intercellular space of the epithelium in NMOSD patients. **(F)** High-power photos showing three types of intercellular junctions in NMOSD patients from the square box of panel **(E)**. The dense cytoplasmic materials attaching to either side of junctions were faded (asterisks). **(D)** Quantitative data show that the intercellular space of these junctions was wider in NMOSD patients than in the HC group (**D**, **p < 0.01). The scale bar (5 μm) in **(E)** is equal to that in **(A)**, and the scale bar (1 μm) in **(F)** is equal that in **(B,C)**.

Our previous studies have shown a disturbance in the flora of feces of NMOSD patients (7, 8). In the present study, we also found colonic mucosa dysbiosis in NMOSD patients. The harmful bacteria and their toxins may attack the epithelium of the mucosa and damage the intercellular junctions between neighboring epithelial cells. Therefore, in the present study, we employed electron microscopy to investigate morphological changes in the sigmoid mucosa. Under a TEM, we observed three types of intercellular junctions between epithelial cells (Figure 2A). TJs connected two epithelia at the apical region close to the intestinal lumen and had a narrow gap and relatively little accumulation of dense cytoplasmic material along this part of the complex (Figure 2B). AJs had a relatively wide gap and extensive condensation of cytoplasmic fibrils attaching to either side of the junctions (Figure 2B). Desmosomes had wider intercellular spaces than AJs and dense and coarse cytoplasmic fibrils attaching on either side of the junction (Figure 2C, asterisks). Compared to the HC, some TJs of the NMOSD patients were separated, and the dense cytoplasmic materials along either side of these three types junctions showed obvious fading (Figures 2E,F, asterisks). The quantitative analysis data demonstrated that the width of the intercellular space was significantly increased in the NMOSD (47.78 ± 2.90 nm) patients compared with the HC (35.72 ± 2.09 nm) subjects (*p* < 0.01) (Figure 2D).

#### Protein Expression of Intercellular Junctions Decreased in the Colonic Mucosa of NMOSD Patients

**FIGURE 3:**
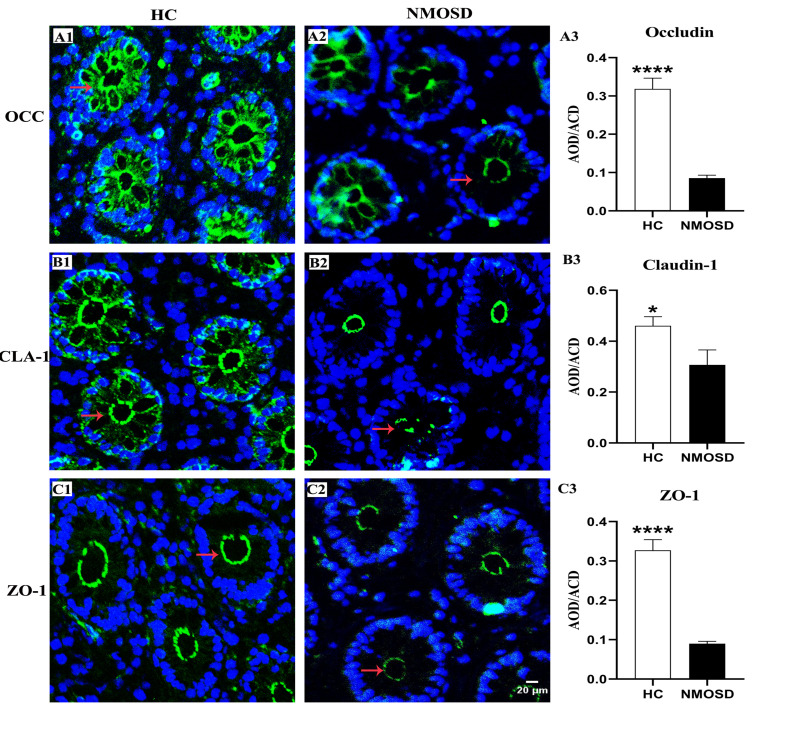
Photomicrograph and histogram showing changes in TJ proteins of the colonic mucosa. Positive immunostaining (arrows, green) for OCC, CLA and ZO-1 of the sigmoid colonic mucosa in the HC and NMOSD groups is shown in **(A1,A2)**, **(B1,B2)**, and **(C1,C2)**, respectively. The nuclei of the epithelium were counterstained with DAPI (blue). The results of the quantitative analysis of OCC, CLA, and ZO-1 are shown in **(A3, B3, and C3)**, respectively. *p < 0.05, ****p < 0.0001 compared with the control group. OCC, occludin; CLA, claudin-1; ZO-1, zonula occludens-1; AOD, average optical density. The scale bar (20 μm) in **(C2)** is equal to that in **(A1,A2)**, **(B1,B2)**, and **(C1)**.

The OCC, CLA, and ZO-1 proteins are important for the integrity and permeability of not only TJs but also AJs (31), and ZO-1 may also be associated with desmosomes (32). Therefore, we used immunofluorescence staining methods to investigate the expression of these proteins in different groups. Photomicrographs showed positive staining for OCC, CLA, and ZO-1 (Figure 3, green). In the HC group, OCC and CLA were distributed at the epithelial surface and intercellular space between epithelia of the colonic mucosa (Figures 3A1,B1, arrows). However, the expression of these two proteins in the intercellular space was barely detected, and only weak positive signals were found in the epithelial surface of the colonic mucosa (Figures 3A2,B2, arrows) in the NMOSD group. Interestingly, ZO-1 was expressed only on the epithelial surface and the intercellular space, even in the HC group (Figure 3C1, arrow), and weak positive signals were observed in NMOSD patients (Figure 3C2, arrows). Consistent with the observations under confocal microscopy, quantitative analyses showed a dramatic decrease in OCC levels in the NMOSD (0.0854 ± 0.0772) group compared to the HC group (0.3185 ± 0.0281), both *p* < 0.01 (Figure 3A3). The expression of CLA in the NMOSD (0.2778 ± 0.0471) group was also decreased compared with that in the HC group (0.4609 ± 0.0353), both *p* < 0.01 (Figure 3B3). Similarly, the ZO-1 level in the NMOSD (0.0899 ± 0.0059) group was also lower than that in the HC group (0.3271 ± 0.0270), both *p* < 0.01 (Figure 3C3).

### Activation of Inflammatory Cells in NMOSD Patients

**FIGURE 4:**
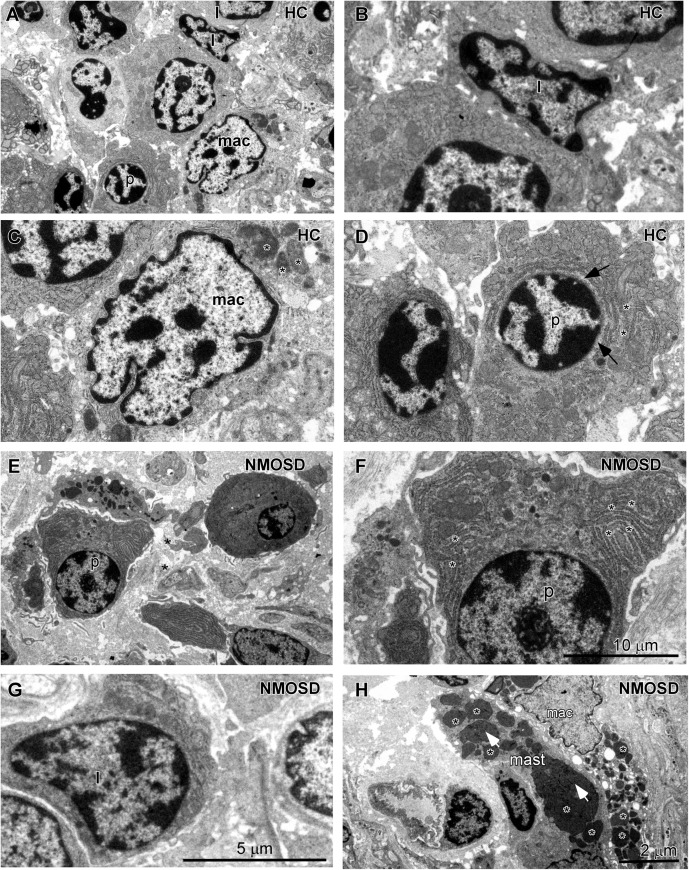
TEM photos showing changes in the morphology of inflammatory cells in the lamina propria of the colonic mucosa between subjects from two groups. **(A)** Different types of cells in the HC subjects included lymphocytes (l), macrophages (mac), and plasma cells (p). **(B)** High-power photo showing a representative lymphocyte (l) from panel **(A)**. **(C)** High-power photo showing a representative macrophage (mac) from panel **(A)**. **(D)** High-power photo showing a representative plasma cell (p) from panel **(A)**. **(E)** Representative larger plasma cells (p) with rich cytoplasm surrounded by two macrophages in an NMOSD specimen. **(F)** High-power photo showing the plasma cell (p) from panel **(E)**. **(G)** A representative larger lymphocyte (l) in an NMOSD specimen. **(H)** A representative macrophage (mac) with a larger number of particles and a mast cell (mast) in the colonic mucosa of an NMOSD patient. The scale bar (10 μm) in **(F)** is equal to that in **(C,D)**. The scale bar (5 μm) in **(G)** is equal to that in **(B)**. The scale bar (2 μm) in **(H)** is equal to that in **(A,E)**.

When the epithelial barrier is damaged, harmful antigens and toxins, such as streptococcal toxins (33), may elicit an inflammatory response from the colonic mucosa. Therefore, we further investigated changes in immune cells of the lamina propria of the colonic mucosa in all subsets of the two groups. Under the TEM, we observed that the normal lymphocytes had a dentate-like nucleus with abundant dense heterochromatin-forming aggregates close to the membrane and little cytoplasm (Figures 4A,B). Macrophages in HC individuals showed a dentated nucleus with a few inclusions in the cytoplasm (Figures 4A,C, asterisks). Plasma cells had a spherical nucleus surrounded by a pale zone (Figures 4A,D, arrows) with moderate rough endoplasmic reticulum surrounding the nucleus (Figures 4A,D, asterisks). For NMOSD patients, we found that plasma cells were larger with well-developed rough endoplasmic retinaculum in the cytoplasm (Figures 4E,F, asterisks). We found that many plasma cells were surrounded by macrophages and were contacted closely by a narrow gap (Figure 4E, arrows). In addition, we also observed some particles appearing in the cytoplasm of the plasma cells (Figure 4F, arrows). Some lymphatic cells were also larger with a larger nucleus (Figure 4G). Many macrophages were burdened with a large number of particles of different sizes (Figure 4H, asterisks). In addition, we found more mast cells, which were armed with larger and dense granules (Figure 4H, asterisks).

**FIGURE 5:**
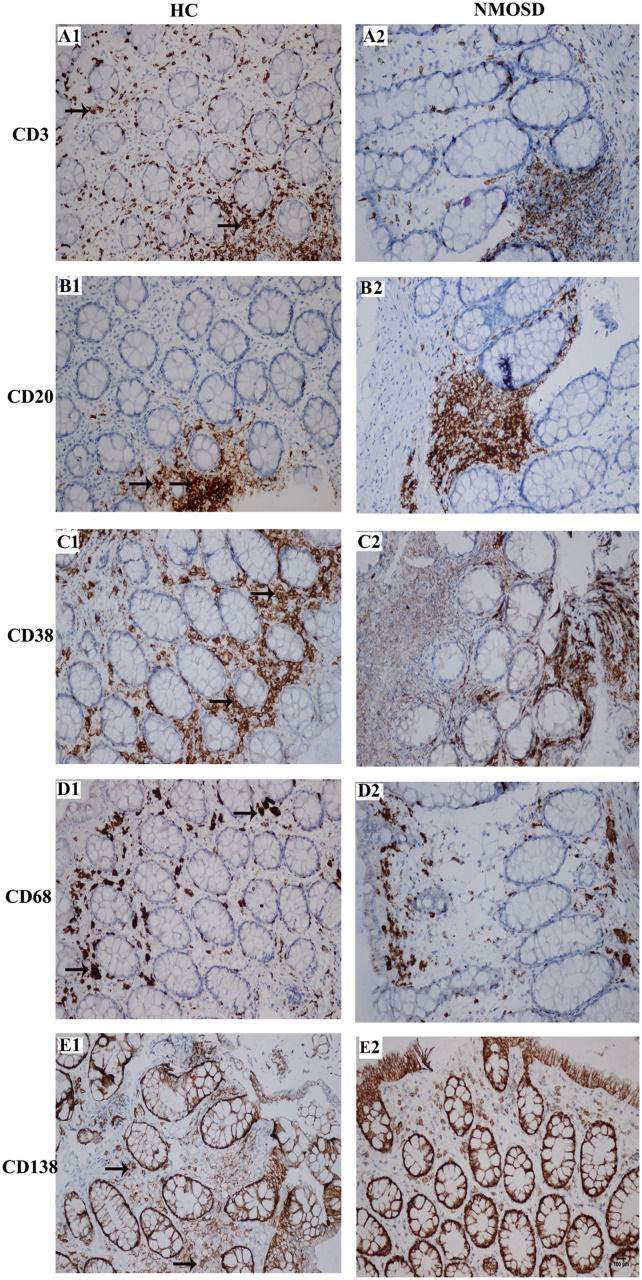
Photomicrographs showing the immunohistochemical staining for inflammatory cell markers using the DAB visualization method. These markers include CD3 [a marker for T cells, **(A1,A2)**], CD20 [a marker for B cells, **(B1,B2)**], CD38 [markers for activated lymphocytes and plasmocytes, **(C1,C2)**], CD68 [a marker for macrophages, **(D1,D2)**] and CD138 [a marker for plasmocytes, **(E1,E2)**]. Positive staining is brown in color (black arrows). All slides were counterstained with hematoxylin (blue). The scale bar (100 μm) in **(E2)** is equal in all photos.

When stimulated by antigens such as bacteria or metabolites, immune cells will respond by increasing the number of cells or increasing the sizes of cells to produce more antibodies. Therefore, we employed immunohistochemistry staining to detect changes in the density and size of inflammatory cells with five antibodies. As shown in Figure 5, the columns represent the HC and NMOSD groups, and the five rows represent CD3-positive cells (T lymphocytes, Figures 5A1,A2), CD20-positive cells (B lymphocytes, Figures 5B1,B2), CD38-positive cells (B lymphocytes and plasma cells, Figures 5C1,C2), CD68-positive cells (monocytes and macrophages, Figures 5C,D1,D2), and CD138-positive cells (plasma cells, Figures 5E1,E2).

**FIGURE 6:**
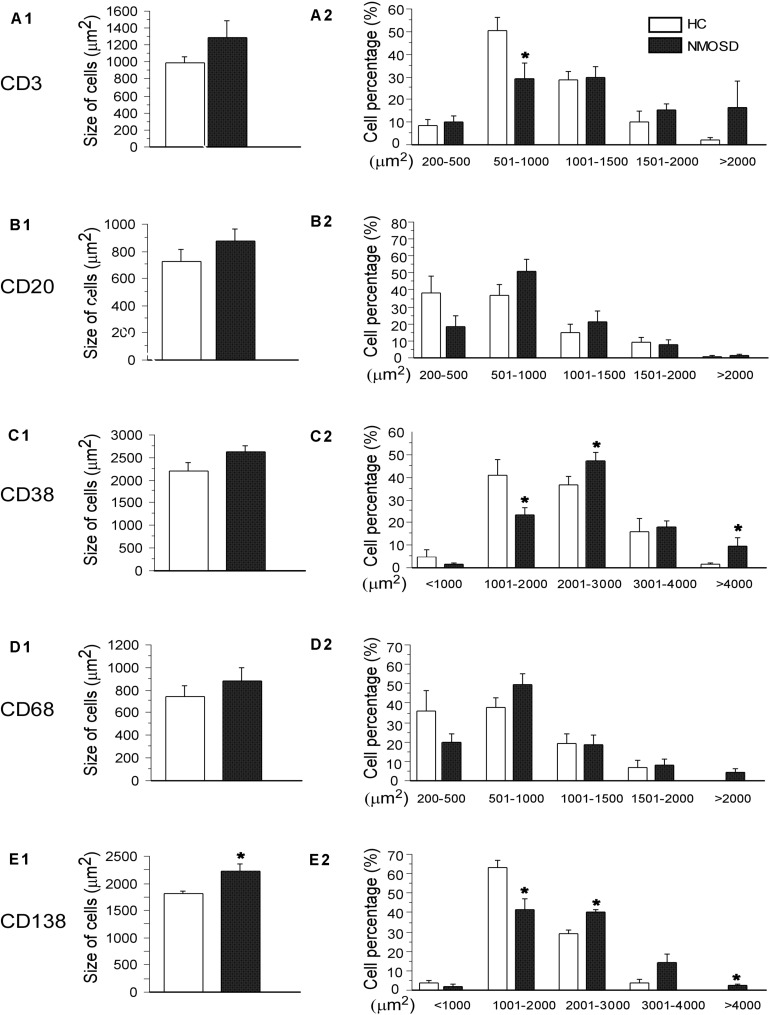
Histogram showing changes in the mean size and the proportion of different size of immunological cells. The left column indicates the mean area of CD3-, CD20-, CD38-, CD68-, and CD138-positive cells **(A1–E1)**, and the right column shows the percentages of different sizes of these cells in the left column **(A2–E2)**. *p < 0.05, compared with the control group.

We counted the numbers of these five types of cells on high-power photos. Unfortunately, no significant difference was found among subjects in the two groups (Supplementary Figure S2). To obtain more detailed and more accurate information from these immunostaining-positive cells, we randomly selected 30–50 cells of each type in each group to draw their areas by ImageJ. From Figures 6, we found a trend where the mean area of each type of cell increased in NMOSD patients, but only CD138-positive cells were markedly increased in the NMOSD group (2225 ± 149 μm^2^) compared with the HC group (1817 ± 40 μm^2^), *p* < 0.05 (Figures 6A1–E1). When we separated the cells into different levels based on the size of the area, we found that the percentage of small (500–1000 μm^2^) CD3-positive cells in the NMOSD (29.0% ± 7.2) group was significantly lower than that in the HC group (50.6% ± 5.5), *p* < 0.05, while the percentage of larger (>2000 μm^2^) CD3-positive cells in NMOSD patients (16% ± 11.5) was higher than that in HC subjects (1.9% ± 1.5), although no significant difference was detected, *p* > 0.05 (Figure 6A2). However, the percentages of small (1000–2000 μm^2^) CD38-positive cells (23.2.5% ± 3.8) and CD138-positive cells (41.3% ± 5.5) were markedly lower in the NMOSD group than in the HC group (41.0% ± 6.8 and 63.5% ± 3.6, respectively), both *p* < 0.05 (Figures 6C2,E2). In contrast, the percentages of larger (2000–3000 μm^2^) CD38-positive cells in NMOSD (47.5% ± 3.3) vs HC (36.5% ± 3.8) subjects and of CD138-positive cells in NMOSD (40% ± 1.7) vs HC (29.1% ± 1.8) subjects were significantly increased, both *p* < 0.05. The percentage of the largest (>4000 μm^2^) CD38-positive cells in the NMOSD vs HC group was 13% ± 4.4 vs 1.0% ± 0.6, and the percentage of the largest CD138-positive cells was 2.3% ± 1.0 vs 0% ± 0, both *p* < 0.05 (Figures 6C2,E2). However, no significant difference was found between each size of CD20- and CD68-positive cells (Figures 6B2,D2).

## Discussion

To the best of our knowledge, the present study is the first to investigate the colon mucosal microbiota and the pathology of the mucosa in patients with NMOSD. Based on the evidence showing a reduction in microbial diversity but abundant *Streptococcus* in the feces of patients with NMOSD from our previous study (8), we further found that the dominant *Granulicatella* and *Streptococcus* species were also present in the colonic mucosa of patients with NMOSD in the present study. Therefore, the evidence from two separate studies showing that decreased bacterial diversity and dominant *Streptococcus* in the feces and colonic mucosa of Chinese patients with NMOSD appears to be consistent. However, a team from University of California reported that *Clostridium perfringens* and *C. perfringens* were enriched taxa in patients with NMOSD and found T helper 17 cells in these patients, which recognize the immunodominant AQP4 epitope and proliferate in response to the corresponding *C. perfringens* ABC-TP peptide, and these two types bacteria were considered participants in the pathogenesis of NMOSD (34, 35). Microbiota diversity is regulated by factors such as host genotype, gender, vaginal or C-section birth, age, environmental non-food conditions, stressors, drugs, diseases, and dietary habits (36–38). The precise reason for the difference in changes in the intestinal microbiota of NMOSD patients reported by our team and University of California’s team seems related to dietary habits.

*Granulicatella* species, along with the genus *Abiotrophia*, were originally known as nutritionally variant Streptococci (NVS), which form part of the normal flora of the oral cavity, genitourinary tract, and intestinal tract. However, they have been associated with a variety of invasive infections in humans such as bacterial endocarditis (39, 40). Streptococci belong a genus of Gram-positive bacteria that are widely distributed across the normal flora of humans and animals and are divided into different species, many of which have the potential to cause invasive infections resulting from the presence of bacteria in a normally sterile site, such as pneumonia, meningitis, and endocarditis (41). *Streptococcus* and Streptococcal toxins have been reported to disrupt cytoplasmic integrity, break down mucocutaneous resistance and cause inflammation (42, 43). Although the effects of *Streptococcus* on the pathogenesis of NMOSD have not been uncovered, more *Streptococcus* in the colonic mucosa may damage the integrity of the epithelium. However, other proinflammatory bacteria which are also abundant in NMOSD in the present study, such as *Proteus* and *Desulfovibrio*, and other toxins such as LPS (44), the immune system such as Th17 cells (45) and components of the diet (46) may also participate in the pathological process.

Aquaporin similarity has been found also between food (vegetables as corn, soybean, spinach, tomato) and brain (47). Furthermore, the vegetables reported above contain, besides aquaporin, also lectins. Dietary lectins may be involved in autoimmune diseases (48) and may regulate inflammation and the expression of matrix metalloproteinases (MMPs) in glial cells (49). Thus, lectins could also cooperate in the inflammatory process.

Epithelial cells join together tightly by different types of intercellular junctions such as TJs, AJs, and desmosomes (50, 51). Additionally, gap junction proteins such as OCC, CLA, and ZO-1 are important in maintaining the integrity of the intestinal epithelium. Defects in ZO-1, OCC, and CLA expression may lead to abnormal intestinal mucosal barrier permeability by increasing paracellular permeability (50, 52). In the present study, we analyzed three types of gap junction proteins (OCC, CLA, and ZO-1), and the results showed that the levels of these proteins were significantly decreased in NMOSD patients. The TEM data also showed that the intercellular spaces in these patients were significantly widened, which may be related to a decrease in gap junction proteins.

When the mucosal barrier is interrupted, potentially pathogens such as microbes, undigested food, endotoxins can induce inflammatory responses in the second defense layer, the lamina propria (53). The lamina propria is armed with different types of immune cells, such as T and B lymphatic cells, plasma cells, mast cells, and macrophages. When B lymphocytes are stimulated by antigens such as viruses or bacteria and metabolites, they transform into plasma cells to produce and release immunoglobulins (54). Plasma cells have abundant cytoplasm with rich rough endoplasmic reticulum and immunoglobulins. Lymphocytes and plasma cells become larger when they are reactivated by producing more antibodies (55). In the present study, although we did not find a difference in the numbers of these cells, we found that the percentages of larger CD38- and CD138-positive cells (plasma cells) increased, suggesting that the number of active plasma cells is increased in the colonic mucosa of NMOSD patients. The TEM data also demonstrated that more larger plasma cells with abundant rough endoplasmic reticulum in the cytoplasm were present in the patients with NMOSD. In addition, the population of large CD68-positive cells (macrophages) with many inclusions also increased, indicating a process of phagocytosis of macrophages and inflammation in the lamina propria. Macrophages and T helper cells participate in the antigen-presenting process from which B lymphocytes are activated and transform into plasma cells (56). In addition, we also found many mast cells with a large number of particles in their cytoplasm in the colonic mucosa of the NMOSD patients. According to recent studies, mast cells are key factors in brain inflammation and NMOSD. These cells can migrate to the CNS through blood vessels and reside on the abluminal side of vessels where they can communicate with neurons, glial cells, and endothelial cells (57). Mast cells have been reported to release a large amount of proinflammatory molecules such as tumor-necrosis factor alpha (TNFα) and interleukins 1β, 4, 5, 6, and 8 (IL-1β, IL4, IL5, IL6, and IL8) (58–60). These molecules not only damage TJs but also induce systemic proinflammatory immune responses (61). However, the precise effect of mast cells on the pathogenesis of NMOSD is still unclear.

Approximately 100 trillion bacteria are present in the intestine along with abundant fungi and viruses, and the intestinal immune system is constantly exposed to microbial antigens, which may serve as stimuli that prolong inflammatory responses (4). Under normal circumstances, translocating pathogens will be endocytosed within the lamina propria and mesenteric lymph nodes (33). However, if the host mucosal immune system is compromised, these defense mechanisms may fail, thus permitting the evasion and survival of bacteria at distant, extraintestinal sites (62, 63). Perturbed gut integrity and permeability may allow pathogens translocate into the circulation, which can increase the host’s susceptibility to various types of diseases by inducing chronic or acute inflammatory responses (3). The persistence of gut-derived molecules and cells in the proximity of the BBB may cause it breakdown, and other elements escaping from the inflamed gut can also impair the integrity of the BBB, such as Th17 (64). Several studies have shown increased BBB permeability in most NMOSD patients (65, 66), and breakdown of the BBB is closely linked to damage to the gut barrier (4, 67, 68).

A recent study reported a case with refractory NMOSD who demonstrated a massive enhancement of cytotoxic behavior in lymphocytes, either in peripheral blood and cerebrospinal fluid (69). Considering the results of the present study and others, destructions of the excessive inflammation of the gut might contribute to the pathology of the NMOSD. Further studies are necessary to accurately reveal the mechanism of the NMOSD underlying intestinal dysbiosis.

Several limitations existed in the present study. First, the number of patients with NMOSD was small. Based on the low incidence of the NMOSD (70), obtaining more colonic mucosa specimens from patients with NMOSD is difficult. We clarified that specimen collection is an invasive procedure with risks of intestinal bleeding and leakage and ensured that the patients were willing to have a piece of colonic mucosa removed for the present study. Second, although we analyzed changes in the expression of gap junction proteins and the intercellular space by morphological methods, alterations of the molecules and functions of gap junctions must be detected by other methods such as western blotting and the lactulose mannitol test to determine whether the integrity of intercellular junctions is interrupted. Third, the present study could not explain the causal relationship between intestinal dysbiosis and intestinal barrier disruption in the NMOSD. Therefore, a well-designed study should be carried on in the future.

## Conclusion

In conclusion, the present study demonstrated that the intestinal barrier was disrupted in the patients with NMOSD, accompanied by dysbiosis and inflammatory activation of the gut. The mucosal microbiota imbalance and inflammatory responses might cause pathogens to cross the damaged intestinal barrier and participate in pathological process in NMOSD. However, further study on the pathological mechanism of NMOSD underlying gut dysbiosis is warranted in the future.

## Data Availability Statement

The raw data supporting the conclusions of this article will be made available by the authors, without undue reservation, to any qualified researcher.

## Ethics Statement

The studies involving human participants were reviewed and approved by The Third Affiliated Hospital of Sun Yat-sen University. The patients/participants provided their written informed consent to participate in this study.

## Author Contributions

CC and ST contributed to the analysis and interpretation of the data, drafting of the manuscript, statistical analysis, and technical and material support. LT was involved in participant enrollment and technical and material support. JG, YC, YW, and PF were involved in technical support and participant enrollment. LT and JG contributed to the analysis and interpretation of the data and technical support. YR was involved in the study concept and design, critical revision of the manuscript, funding procurement, study supervision, and final approval of the version to be published. WQ contributed to study supervision, participant enrollment, and critical revision of the manuscript. All authors read and approved the final version of the manuscript.

## Conflict of Interest

The authors declare that the research was conducted in the absence of any commercial or financial relationships that could be construed as a potential conflict of interest.
