# Supplementary figures and images for: Intestinal Barrier Breakdown and Mucosal Microbiota Disturbance in Neuromyelitis Optical Spectrum Disorders
Source: Front Immunol. 2020 Sep 2;11:2101. doi: 10.3389/fimmu.2020.02101 (PMC7492665; doi:10.3389/fimmu.2020.02101)

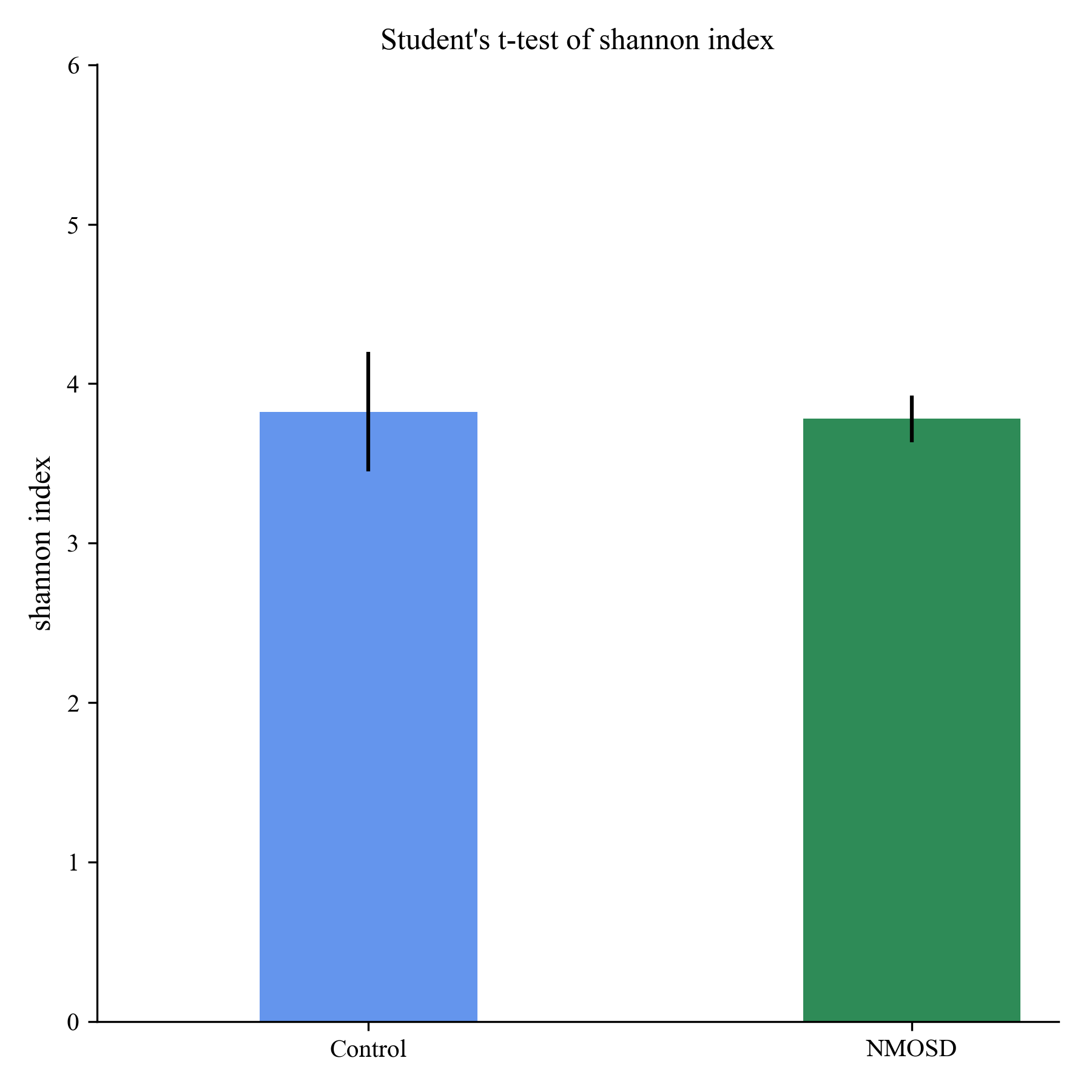

Supplement: Supplementary file 1 [file Image_1.PNG]

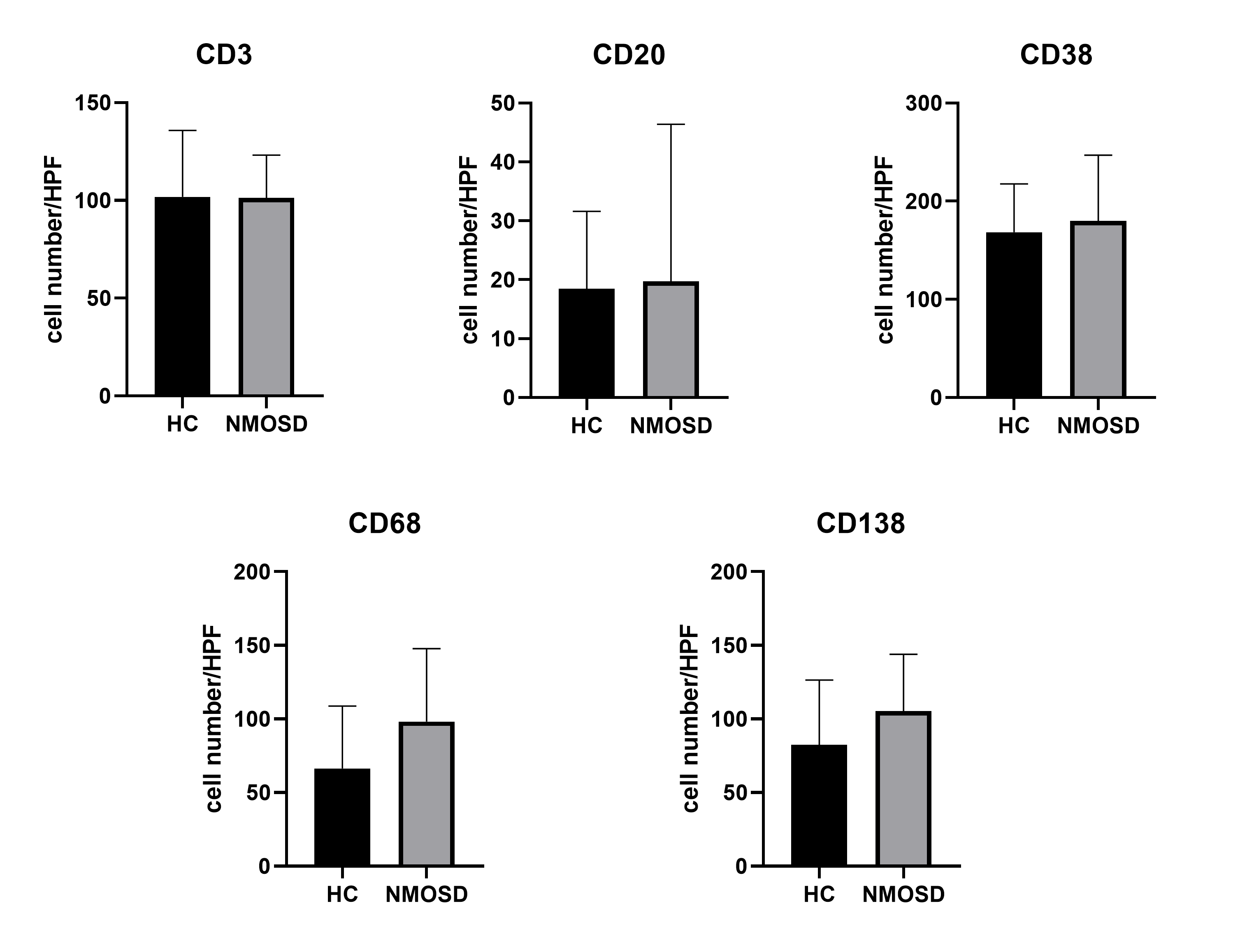

Supplement: Supplementary file 2 [file Image_2.TIF]
